# Inorganic Nitrogen Supply and Dissolved Organic Nitrogen Abundance across the US Great Plains

**DOI:** 10.1371/journal.pone.0107775

**Published:** 2014-09-22

**Authors:** Megan L. Mobley, Matthew J. Cleary, Ingrid C. Burke

**Affiliations:** 1 Department of Botany, University of Wyoming, Laramie, Wyoming, United States of America; 2 Helga Otto Haub School of Environment and Natural Resources, University of Wyoming, Laramie, Wyoming, United States of America; 3 Program in Ecology, University of Wyoming, Laramie, Wyoming, United States of America; University of Illinois at Chicago, United States of America

## Abstract

Across US Great Plains grasslands, a gradient of increasing mean annual precipitation from west to east corresponds to increasing aboveground net primary productivity (ANPP) and increasing N-limitation. Previous work has shown that there is no increase in net N mineralization rates across this gradient, leading to the question of where eastern prairie grasses obtain the nitrogen to support production. One as-yet unexamined source is soil organic N, despite abundant literature from other ecosystems showing that plants take up dissolved soil organic N. This study measured KCl-extractable dissolved organic N (DON) in surface soils across the grassland productivity gradient. We found that KCl-extractable DON pools increased from west to east. If available to and used by plants, this DON may help explain the high ANPP in the eastern Great Plains. These results suggest a need for future research to determine whether, in what quantities, and in what forms prairie grasses use organic N to support primary production.

## Introduction

It has in the past been widely accepted that nitrogen compounds must undergo mineralization – that is, transformation from organic to inorganic forms - to become available to plants [1]. Yet, in a variety of ecosystems, plants have been shown to take up dissolved organic nitrogen (DON) directly from the soil, as reviewed by [2], [3]. These plant-accessible organic N compounds include amino acids, amino sugars, amino-polysaccharides, nucleic acids, and various purine and pyrimidine compounds [2]. A new paradigm concerning N cycling has gained acceptance [2], in which the rate of depolymerization of organic N polymers into monomers by microbial enzymes, i.e., the production of DON, regulates ecosystem N cycling rather than the rate of N mineralization.

The ecological importance of DON acquisition is still unclear in many ecosystems [4], including temperate grasslands. Jones et al. [5], [6] suggest that temperate grassland species can take up DON directly from the soil, although it is unclear how important DON is relative to other sources. Knowledge about the magnitude and importance of plant uptake of DON is important for our understanding and predictions of N cycling using models, and for clarifying the dynamics of plant-microbial competition. Understanding the potential for grassland plant use of DON could lead to management strategies that reduce fertilizer use and associated nutrient runoff and pollution while still supporting productive and N-retentive cereal croplands and biofuel-producing grasslands.

For grasslands of the United States, the combined results of previous research do not frame a coherent understanding of grassland N cycling. A number of studies have shown grassland plant production to respond to experimental N additions, suggesting that these ecosystems are N–limited [7]–[10]; a literature review concluded that although water was an important co-limiter of the semi-arid western grasslands, N-limitation was the rule across the Great Plains [11]. Aboveground net primary production (ANPP) increases with mean annual precipitation from semiarid shortgrass steppe in the west through mixed grass prairie to the subhumid tallgrass prairie in the east [12]. Both the concentration of N in biomass and the litter decomposition rate decrease, and nutrient use efficiency increases, along this precipitation gradient [13]. Nevertheless, plant N uptake, as indicated by the quantity of N in aboveground production (ANPP-N), increases along this precipitation and production gradient [14], [15]. These lines of evidence suggest that the severity of N-limitation increases from west to east.

To explain observed patterns of ANPP, simulations of the Century model predict a linear increase of net N mineralization rates with precipitation [16], yet field studies have reported no west-to-east trend in net N mineralization [14], [15]. Total soil N, on the other hand, has been shown to be higher in tallgrass than shortgrass prairie [13]–[15], [17], suggesting that organic N may play a role in supplying the additional N needed to support eastern tallgrass prairie production. This could explain the discrepancy between model predictions and field studies, as commonly used ecosystem models do not consider the rate of organic N depolymerization or plant uptake of organic N, focusing only on inorganic N mineralization [18]–[21].

This exploratory study examined the spatial pattern in abundances and rates of production of KCl-extractable dissolved inorganic N (DIN) and DON along the west to east precipitation gradient across US grasslands.

## Materials and Methods

### Site Description

The Nature Conservancy, Fort Hays State University, USDA-ARS, and the Long-Term Ecological Research programs at Konza Prairie and Shortgrass Steppe provided access to field sites spanning from eastern Colorado to eastern Kansas [14], [15], [17]. The entire gradient spans precipitation and temperature ranges of 500 mm and 4.4°C, respectively. The two westernmost sites, Shortgrass Steppe (SGS) Long-Term Ecological Research (LTER) and Arikaree River (ARI) Nature Conservancy site, are located in the drier shortgrass steppe. Two sites, Smokey River (SVR) Nature Conservancy site and a property of Ft. Hays State University (HAYS), were located in the northern mixed grass prairie. The easternmost site was located in the wetter tallgrass prairie, Konza Prairie LTER (KON) (Table 1). Each sampling location was located on native upland grasslands with moderate grazing by cattle in the summer and minimal disturbance. The soils at all sites ranged from loam to sandy loam. The vegetation of the shortgrass steppe was dominated by *Bouteloua gracilis* (Willd. ex Kunth) Lag. ex Griffiths and *Buchloë dactyloides* (Nutt.) J.T. Columbus, whereas *Andropogon gerardii* Vitman and *Schizachyrium scoparium* (Michx.) Nash dominated the tallgrass prairie. The mixed-grass prairie comprised a combination of all of the species above.

**Table 1:** Locations and attributes of grassland sites.

	SGS:	ARI:	SVR:	HAYS:	KON:
	Shortgrass Steppe LTER	Arikaree River TNC	Smokey River TNC	Ft. Hays State Univ.	Konza Prairie LTER
Latitude, Longitude	40°48.8′N, 104°42.8′W	39°45.4′N, 102°26.9′W	38°53.6′N, 100°58.7′W	38°52.4′N, 99°23.3′W	39°4.8′N, 96°33′W
MAP (mm)	320	450	502	575	835
MAT (°C)	8.6	9.7	10.8	11.9	13.0
Grassland Type	Shortgrass Steppe	Shortgrass Steppe	Mixed Grass Prairie	Mixed Grass Prairie	Tallgrass Prairie
ANPP-N [15] (g N m^−2^ y^−1^)	1.1	1.7	1.5	1.6	2.4
ANPP-C [15] (g C m^−2^ y^−1^)	143	123	221	211	337
Net N min. [14] (g N m^−2^ y^−1^)	1.5 (0.49)	3.0 (1.04)	1.5 (0.53)	3.0 (0.63)	2.4 (0.26)
Soil C (g C m^−2^)	1367 (67.9)	1440 (68.6)	1851 (52.9)	5204 (155.4)	2373 (151.2)
Soil N (g N m^−2^)	287.2 (13.1)	274.2 (13.0)	214.6 (13.3)	285.1 (5.9)	210.7 (11.0)
Soil C:N	4.91 (0.31)	5.33 (0.25)	9.05 (0.50)	18.3 (0.44)	11.2 (0.32)
Volumetric Soil Moisture (%)	13.8 (0.40)	7.3 (0.53)	3.7 (0.17)	3.4 (0.36)	6.3 (0.30)

MAP =  mean annual precipitation (mm), MAT =  mean annual temperature (°C), TNC =  The Nature Conservancy, LTER =  National Science Foundation Long-Term Ecological Research site. Data in lower panel is from this study; values in parentheses represent one standard error of the mean.

### Field Sampling

At each site, we set up three 25 m long transects within a 100 m radius of each other in undisturbed areas representative of the surrounding vegetation. From June 6–15, 2011, we collected soil cores (10 cm length ×5 cm diameter) from two microsites (under plant canopies and interspaces between plants) and at two depths (0–5 cm and 5–10 cm) at three random locations on each transect, for a total of 180 soil samples (2 microsites ×2 depths ×3 locations ×3 transects ×5 sites). Soils were immediately sieved through a 2 mm mesh and homogenized in a plastic bag.

We extracted soils immediately for dissolved N pools, in order to minimize disturbance-induced N transformations associated with delays in processing, and to obtain the most realistic estimates of DON and DIN concentrations possible [22]. We extracted dissolved pools of N following the procedure outlined by Robertson et al. [23] and Bardgett et al. [24]. A 10 g subsample of field-fresh, sieved, homogenized soil was placed in an extraction cup with 100 mL 2M KCl solution and shaken for 10 minutes. The extraction cups were returned to the laboratory at the University of Wyoming, Laramie, WY, to be filtered and frozen within two days of sampling. The remaining soil was air-dried and stored in paper bags until further analysis.

We installed one pair of Plant Root Simulator (PRS) resin probes (Western Ag Innovations Inc., Saskatoon, Saskatchewan) per microsite at 10 random locations along each transect (2 microsites ×10 locations ×3 transects ×5 sites  = 300 pairs), to estimate inorganic N supply rate. Each pair consisted of one anion and one cation probe to collect mobile NO_3_^−^ and NH_4_^+^, respectively. Probes were inserted into the soil with the adsorption membrane extending from the surface to 5.5 cm depth, and were buried for 30 days from early May to early June 2011. At each site, the probes were removed on the same day as the soil sampling. Probes were sorted into two groups of five pairs of probes from each microsite type. This resulted in two replicate sets of probes per each microsite type per each transect for a total of 60 (2 replicates ×2 microsites ×3 transects ×5 sites) probe sets. Probes were washed in the laboratory with DI water within two days of unearthing and then sent to Western Ag Innovations, Inc. (Saskatoon, Saskatchewan) for inorganic N analysis.

### Laboratory Analyses

The eluate from the extraction cups was shaken for 1 hour and filtered in the laboratory using Whatman #1 filter paper and frozen immediately thereafter. For measurement of total KCl-extractable dissolved total N and inorganic N, we thawed the eluate and diluted a 2.5 mL subsample with 17.5 mL DI water. We analyzed total dissolved N on a Shimadzu TOC-V Analyzer (Shimadzu Scientific Instruments, Wood Dale, IL, USA). Dissolved inorganic N was analyzed at the Natural Resource Ecology Laboratory (Colorado State University, Fort Collins, CO) using an Alpkem Flow Solution IV Automated wet chemistry system (O.I. Analytical, College Station, TX, USA). We estimated KCl-extractable DON as the difference between total dissolved N and dissolved inorganic N.

We mixed and subsampled remaining air-dried soils for analysis of total C and N. Subsamples were ground on a rotary soil grinder (Planetary Micro Mill Pulverisette 7, Fritsch GmbH, Idar-Oberstein, Germany), and 2 mg of ground soil were analyzed on a Perkin Elmer 2400 Elemental Analyzer (Perkin Elmer, Inc., Waltham, MA, USA) for total C and N. Volumetric water content was estimated after drying a 100 g subsample of field moist soil at 105°C for one day [23].

### Statistical Analyses

We performed all analyses in the statistical program R, version 2.14.2 [25]. Extractable DIN and DON concentrations were multiplied by the soil sample depth (5 cm) and bulk density (mean  = 0.877 g cm^−3^) to obtain DIN and DON pools on a per-area basis. Data for the two soil depth increments (0–5 cm, 5–10 cm) were aggregated such that all analyses were of soil properties and constituents to 10 cm depth, with the exception of PRS probe data that characterized the top 5 cm of soil. We employed two-way ANOVAs coupled to Tukey multiple comparison tests [26] to test differences in the dependent variables total soil C (g C m^−2^), N (g N m^−2^), C:N (mass C:N), and 0–5 cm soil inorganic N supply rate (µg N 10 cm^−2^ month^−1^) among sites and between microsites. Two-way ANCOVAs tested differences in the dependent variables total soil KCl-extractable DIN (g DIN m^−2^) and KCl-extractable DON (g DON m^−2^) among sites and between microsites with volumetric soil moisture as a continuous independent variable. One extreme outlier value for DON with strong influence on the ANCOVA (DON>17 g N m^−2^; outlier test Bonferonni *p* = 4.4×10^−26^) was excluded from DON analyses. ANOVAs were called using aov(), with drop1(model, test = “F”) to extract type III sums of squares. Multiple comparisons were performed using TukeyHSD(). We performed linear regressions using lm() to assess the effect of soil moisture on DIN and DON measurements, and to test for a relationship between KCl-extractable DON pools and MAP. A paired *t*-test tested differences in DON and DIN abundance (g N m^−2^) across all sites and microsites. Significance was assessed at *α* = 0.05. Data for this study can be found in the Supporting Information.

## Results

Soil C differed significantly among sites (among-sites *F* = 212, d.f. = 4, *p*<0.0001) and was significantly higher in the tallgrass prairie than shortgrass steppe (*p*<0.0001, Table 1). Total soil N (g N m^−2^) decreased from west to east across the grassland gradient (among-sites *F* = 10.7, d.f. = 4, *p*<0.0001, Table 1). Soil C:N increased significantly from west to east across the gradient (among-sites *F* = 211, d.f. = 4, *p*<0.0001). There were no significant differences in soil C (*p* = 0.439), N (*p* = 0.537), or C:N between under-plant and plant interspace microsites (*p* = 0.101).

**Figure 1:**
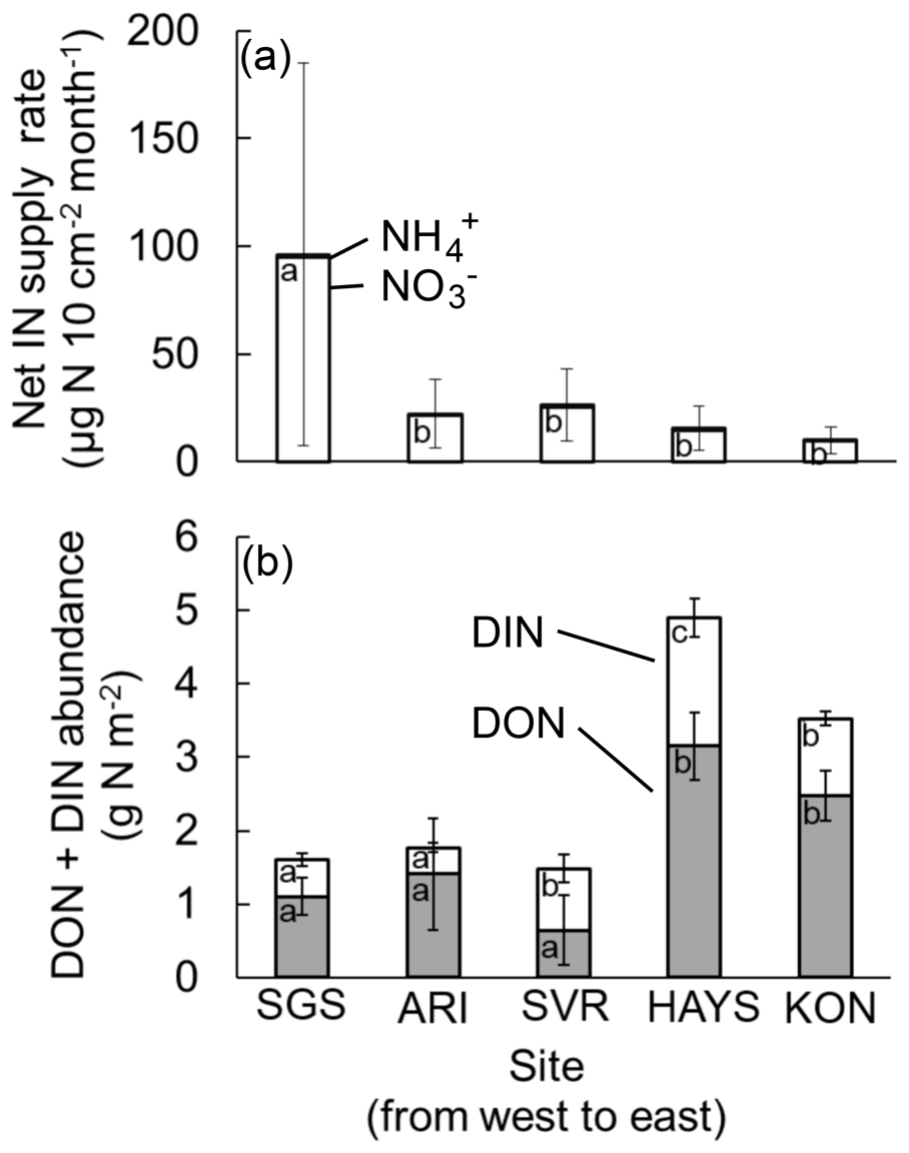
Inorganic N supply rates and extractable dissolved N pools at grassland sites. a) 0–5 cm soil net inorganic N supply rates (NH_4_^+^ in black bars, NO_3_^−^ in white bars) at five grasslands of the US Great Plains, arranged from west to east. Error bars represent 95% confidence intervals on total (NH_4_^+^+NO_3_^−^) inorganic N supply rate. Lowercase letters represent significant site differences at *α* = 0.05. b) Total extractable dissolved soil N (DIN+DON) to 10 cm soil depth across the US Great Plains. Lowercase letters in the gray columns represent significant differences in DON among sites; lowercase letters in the white columns indicate significant differences in DIN among sites. Multiple comparisons were performed on output of ANOVAs that did not include soil moisture as a covariate. Error bars represent 95% confidence intervals. Significance assessed at *α* = 0.05. One extreme outlier (DON>17 g N m^−2^ at SVR) was excluded from the analysis.

The 30-day inorganic N supply rate estimated with PRS probes was highly variable and showed a slight decline across the gradient, with the supply rate at the easternmost site significantly lower than that of the westernmost site (among-sites *F* = 8.07, d.f. = 4, *p*<0.0001; Figure 1a). There were no significant differences in inorganic N supply rates between microsites.

**Figure 2:**
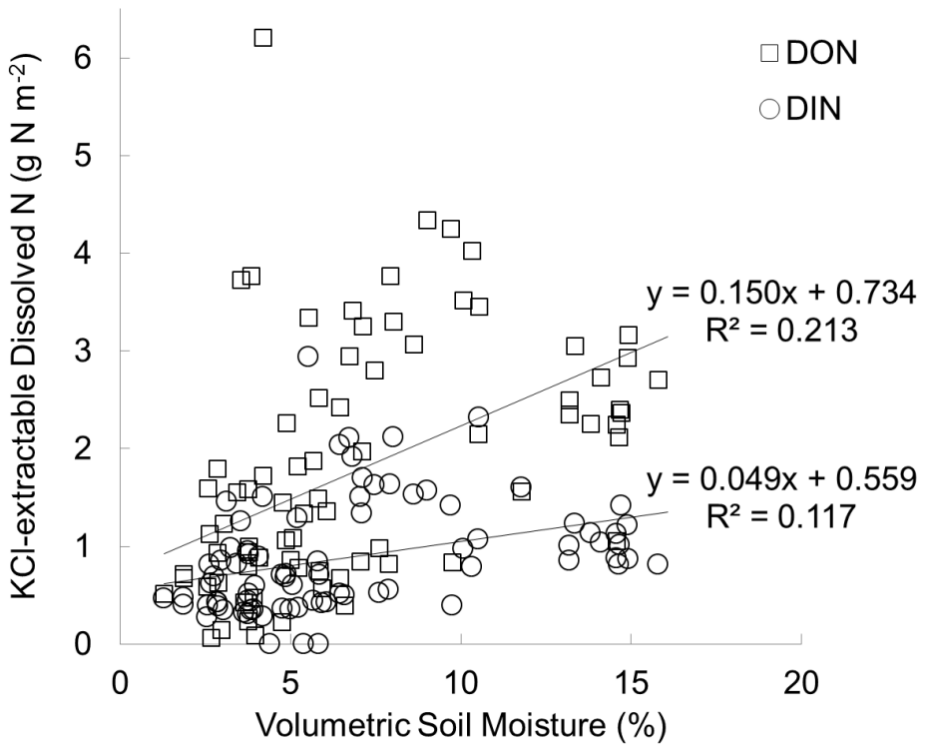
Simple linear regressions of KCl-extractable soil dissolved inorganic (DIN; circles) and organic nitrogen (DON; squares) against volumetric soil moisture for 0–10 cm soil samples from five sites across the Great Plains.

The pool of KCl-extractable dissolved inorganic nitrogen (DIN) was greater in the eastern tallgrass prairie compared to the western shortgrass steppe (among-sites *F* = 44.9, d.f. = 4, *p*<0.0001; Figure 1b). KCl-extractable dissolved organic N was significantly more abundant in the tallgrass region compared to the shortgrass steppe (among-sites F = 10.6, d.f. = 4, p<0.0001; Figure 1b). There were no significant differences in DIN (*p* = 0.128) or DON (*p* = 0.461) between microsites. Across all sites, abundances of KCl-extractable DON were significantly higher than DIN (paired *t* = 6.648, d.f. = 82, *p*<0.0001). Both DIN (F = 10.4, d.f. = 79, R^2^ = 0.106, p = 0.0018) and DON (F = 21.1, d.f. = 78, R^2^ = 0.202, p<0.0001) increased with soil moisture (Figure 2), but soil moisture was not significant as a covariate of DIN (*p* = 0.277) or DON (*p* = 0.241) in the ANCOVAs. There was a positive relationship between DON measurements and site-level ANPP (F = 6.257, d.f. = 42, R^2^ = 0.130, *p* = 0.0164; Figure S1).

## Discussion

This study tested the hypothesis that extractable dissolved organic N pools would increase along a precipitation and production gradient from west to east across US grasslands. Unlike two previous studies at the same sites [14], [15], we did not find a west to east increase in total soil N; soil C and C:N did increase from west to east as in previous studies. Also like previous studies, we found no increase in inorganic N supply rates from west to east, even though plant uptake does increase (ANPP-N, Table 1). This supports previous conclusions that N becomes more limiting, and the N cycle grows tighter, from west to east. We did observe, however, that pools of KCl-extractable dissolved inorganic and organic N, representing exchangeable + free soil N [27], increased from west to east.

These data must be considered as suggestive, rather than conclusive, given a number of limitations to this small study. Our one-time measurements of KCl-extractable DIN and DON and our one resin probe incubation give an incomplete characterization of the long-term mean pools and rates for these grassland sites. Pool size is not necessarily indicative of cycling rate or ecological importance, as a large pool might result from high production or low demand. We did not characterize the chemical composition or molecular weight distribution of DON, thus we cannot speculate on how much of the DON is potentially plant-available. There are artifacts associated with each of the field methods that could reduce the sensitivity of N pool and production estimates. The soil and root disturbance associated with soil sampling may release DON that would not naturally be available in the soil [4], and use of KCl as an extractant may result in a pool of extractable dissolved N that is quite different in composition, quantity, and quality than that found under natural conditions [28], [29]. As evidenced by the significant relationships between KCl-extractable N pools and soil moisture (Figure 2), the KCl-extraction likely overestimates what is actually plant-accessible under normal dry soil conditions. Because less nitrate is likely to reach the resin probes when soil moisture is limiting, regardless of actual mineralization rates, we consider resin probes to be better indicators than extractions of the available N that is accessible by plants [30]. These methodological artifacts should not affect comparisons among sites within a given study if consistent methods are used at all sites, as was the case in this study and previous studies at these sites [14], [15].

Alternative or additional sources of ANPP-N may include biological N fixation and atmospheric N deposition. N fixation, largely by free-living organisms, has been estimated at <0.1 g N m^−2^ y^−1^ in shortgrass steppe [31] and ∼0.8 g N m^−2^ y^−1^ in tallgrass prairie [32], although it is unclear how much of that fixed N enters the plant-soil N cycle versus that volatilized or denitrified [33]. Total wet+dry inorganic N deposition increases from approximately 0.35 g N m^−2^ y^−1^ at SGS to approximately 0.71 g N m^−2^ y^−1^ at KON [34], [35], and so could also provide a portion of the additional N required to support ANPP.

This study provides one piece of support for the broader hypothesis that organic N may serve as a nitrogen source for grassland production—a source that would explain how plant N uptake (ANPP-N) is able to increase along the precipitation gradient without a concomitant increase in N mineralization rates. Our results show that a sizeable pool of KCl-extractable dissolved organic N resides in these grassland soils, and that this N pool increases from west to east across the Great Plains precipitation gradient. The larger KCl-extractable DON pools at the eastern end of the precipitation gradient open the possibility that DON as a source of plant N may help resolve discrepancies in between model predictions and measurements of available N. If so, ecosystem models such as Biome-BGC, Century, PnET, and TEM may be missing a source of N for grasslands. These models represent the N required for primary productivity as “mineral N” generated by N mineralization [18]–[21]. If organic N, which these results indicate is abundant at these sites, is important for plant growth, then an important nutrient source is being left out of model simulations.

## Conclusions

We found that the abundance of DON increased across the west-to-east along the precipitation and productivity gradient of US Great Plains grasslands, suggesting that it could be a significant source of N if plants are able to utilize it. If organic N is an important component of available N in US grasslands, it may explain why ecosystem models that do not include plant use of organic N are unable to predict N supply rates across the US grasslands. Additional studies must be conducted to determine if grass species dominating the US Great Plains assimilate biologically-relevant quantities of organic N.

## Supporting Information

Figure S1 **The relationship between KCl-extractable soil DON and ANPP.** Simple linear regression of KCl-extractable soil dissolved organic nitrogen (DON; microsites and depths for each sample location mathematically combined to yield *n* = 9 replicate measurements per site) against site-level mean aboveground net primary production (ANPP; [15]) for five grassland sites across the US Great Plains.
(TIF)

Data S1 **Bulk soil C and N, KCl-extractable N, and inorganic N supply rate data.**
(XLSX)
