# Supplementary figures and images for: Inorganic Nitrogen Supply and Dissolved Organic Nitrogen Abundance across the US Great Plains
Source: PLoS One. 2014 Sep 22;9(9):e107775. doi: 10.1371/journal.pone.0107775 (PMC4171503; doi:10.1371/journal.pone.0107775)

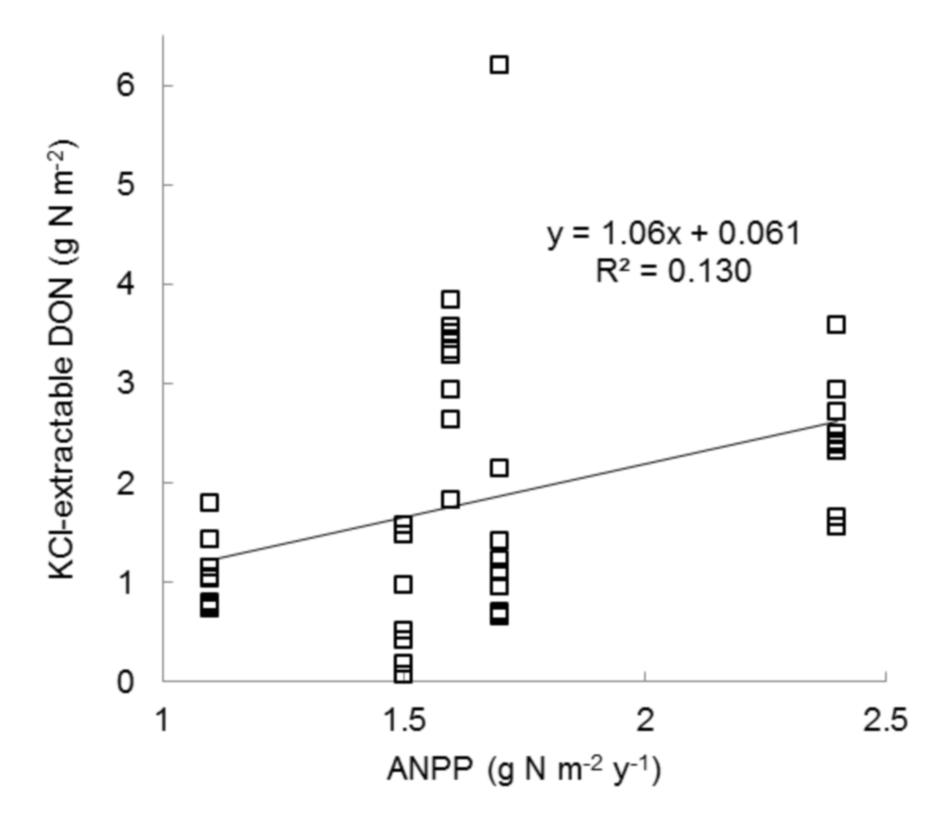

Supplement: Figure S1 — The relationship between KCl-extractable soil DON and ANPP. Simple linear regression of KCl-extractable soil dissolved organic nitrogen (DON; microsites and depths for each sample location mathematically combined to yield n = 9 replicate measurements per site) against site-level mean aboveground net primary production (ANPP; [15]) for five grassland sites across the US Great Plains. (TIF) [file pone.0107775.s001.tif]
